# Prediction of Cardiovascular Parameters With Supervised Machine Learning From Singapore “I” Vessel Assessment and OCT-Angiography: A Pilot Study

**DOI:** 10.1167/tvst.10.13.20

**Published:** 2021-11-12

**Authors:** Louis Arnould, Charles Guenancia, Abderrahmane Bourredjem, Christine Binquet, Pierre-Henry Gabrielle, Pétra Eid, Florian Baudin, Ryo Kawasaki, Yves Cottin, Catherine Creuzot-Garcher, Sabir Jacquir

**Affiliations:** 1 Ophthalmology Department, University Hospital, Dijon, France; 2 INSERM, CIC1432, Clinical Epidemiology Unit, Dijon, France; Dijon University Hospital, Clinical Investigation Center, Clinical Epidemiology/Clinical Trials Unit, Dijon, France; 3 Centre des Sciences du Gout et de l'Alimentation, AgroSup Dijon, CNRS, INRAE, Université Bourgogne Franche-Comté, Dijon, France; 4 Cardiology Department, University Hospital, Dijon, France; 5 PEC 2, University Hospital, Dijon, France; 6 Department of Vision Informatics, Osaka University Graduate School of Medicine, Suita, Japan; 7 Université Paris-Saclay, CNRS, Institut des Neurosciences Paris-Saclay, Gif-sur-Yvette, France

**Keywords:** retina, supervised machine learning (ML), cardiovascular risk score, optical coherence tomography angiography (OCT-A)

## Abstract

**Purpose:**

Assessment of cardiovascular risk is the keystone of prevention in cardiovascular disease. The objective of this pilot study was to estimate the cardiovascular risk score (American Hospital Association [AHA] risk score, Syntax risk, and SCORE risk score) with machine learning (ML) model based on retinal vascular quantitative parameters.

**Methods:**

We proposed supervised ML algorithm to predict cardiovascular parameters in patients with cardiovascular diseases treated in Dijon University Hospital using quantitative retinal vascular characteristics measured with fundus photography and optical coherence tomography – angiography (OCT-A) scans (alone and combined). To describe retinal microvascular network, we used the Singapore “I” Vessel Assessment (SIVA), which extracts vessel parameters from fundus photography and quantitative OCT-A retinal metrics of superficial retinal capillary plexus.

**Results:**

The retinal and cardiovascular data of 144 patients were included. This paper presented a high prediction rate of the cardiovascular risk score. By means of the Naïve Bayes algorithm and SIVA + OCT-A data, the AHA risk score was predicted with 81.25% accuracy, the SCORE risk with 75.64% accuracy, and the Syntax score with 96.53% of accuracy.

**Conclusions:**

Performance of these algorithms demonstrated in this preliminary study that ML algorithms applied to quantitative retinal vascular parameters with SIVA software and OCT-A were able to predict cardiovascular scores with a robust rate. Quantitative retinal vascular biomarkers with the ML strategy might provide valuable data to implement predictive model for cardiovascular parameters.

**Translational Relevance:**

Small data set of quantitative retinal vascular parameters with fundus and with OCT-A can be used with ML learning to predict cardiovascular parameters.

## Introduction

Retinal vascular imaging is constantly improving. The retinal microvascular network can be thoroughly described with fundus photograph imaging analysis software, such as the Singapore “I” Vessel Assessment (SIVA).^1^^,^^2^ In addition, the quantitative description of retinal microvascularization was recently enhanced by the development of a new noninvasive technique: optical coherence tomography angiography (OCT-A).^3^ It has been shown that quantitative retinal vascular characteristics in both fundus photographs with SIVA software and metrics of retinal vascular density with OCT-A are associated with patients’ systemic vascular alteration,^4^ cardiovascular risk profile^5^^,^^6^ and cardiovascular complications.^7^ In addition, deep learning algorithms have been described for diabetic retinopathy detection, retinopathy of prematurity screening, grading of age-related macular degeneration, and glaucoma detection.^8^^–^^11^ Furthermore, Poplin et al. recently indicated that retinal imaging analysis of fundus photography using deep learning could be used to predict a wide range of cardiovascular risk factors.^12^ However, deep learning is limited in some healthcare applications, particularly in a context of sparce data and real world clinical data.^13^ Thus, machine learning (ML) methods could be trained more easily and provide better overall performance when compared to deep learning with a small data set. We hypothesized that classic ML algorithms could predict cardiovascular risk scores and systemic parameters from quantitative retinal vascular data obtained with SIVA software and OCT-A. Better cardiovascular risk stratification is consequently of growing interest given that cardiovascular disease (CVD) remains one of the leading causes of death worldwide.^14^ The purpose of this pilot study was to develop a prediction model with a supervised ML approach to cardiovascular parameters using retinal vascular characteristics measured with SIVA software and OCT-A (alone and combined). Our objective was to estimate the cardiovascular risk score (American Hospital Association [AHA] risk score, Syntax score, and SCORE risk) with this ML model.

## Patients and Methods

### Study Design and Patients

This pilot study was an ancillary study of a previous pilot prospective cross-sectional study conducted in Dijon University Hospital's Cardiology Intensive Care Unit. The methodology of the EYE-MI study and the patients’ baseline characteristics have been detailed elsewhere.^5^ Briefly, from May 2016 to May 2017, patients presenting with acute coronary syndrome (ACS) were included. They were taken to the ophthalmology department within the first 2 days of their hospitalization for an examination of the retinal microvasculature using OCT-A and fundus photographs. The exclusion criteria were: retinal disease (vascular occlusion, diabetic retinopathy, and macular degeneration), patients under 18 years of age, those under guardianship, patients with hemodynamic instability, and patients without both retinal examinations (SIVA and OCT-A). We also excluded severely myopic eyes (axial length greater than 26 mm) because this could affect retinal microvascular density.^15^ The study was approved by the Dijon University Hospital ethics committee and was registered as 2017-A02095-48. It complied with the tenets of the Declaration of Helsinki and a written informed consent was obtained from the patients. We followed the Strengthening the Reporting of Observational Studies in Epidemiology (STROBE) statement according to the EQUATOR Guidelines.^16^

### Retinal Microvascular Image Acquisition and Quantitative Analysis

**Figure 1.**
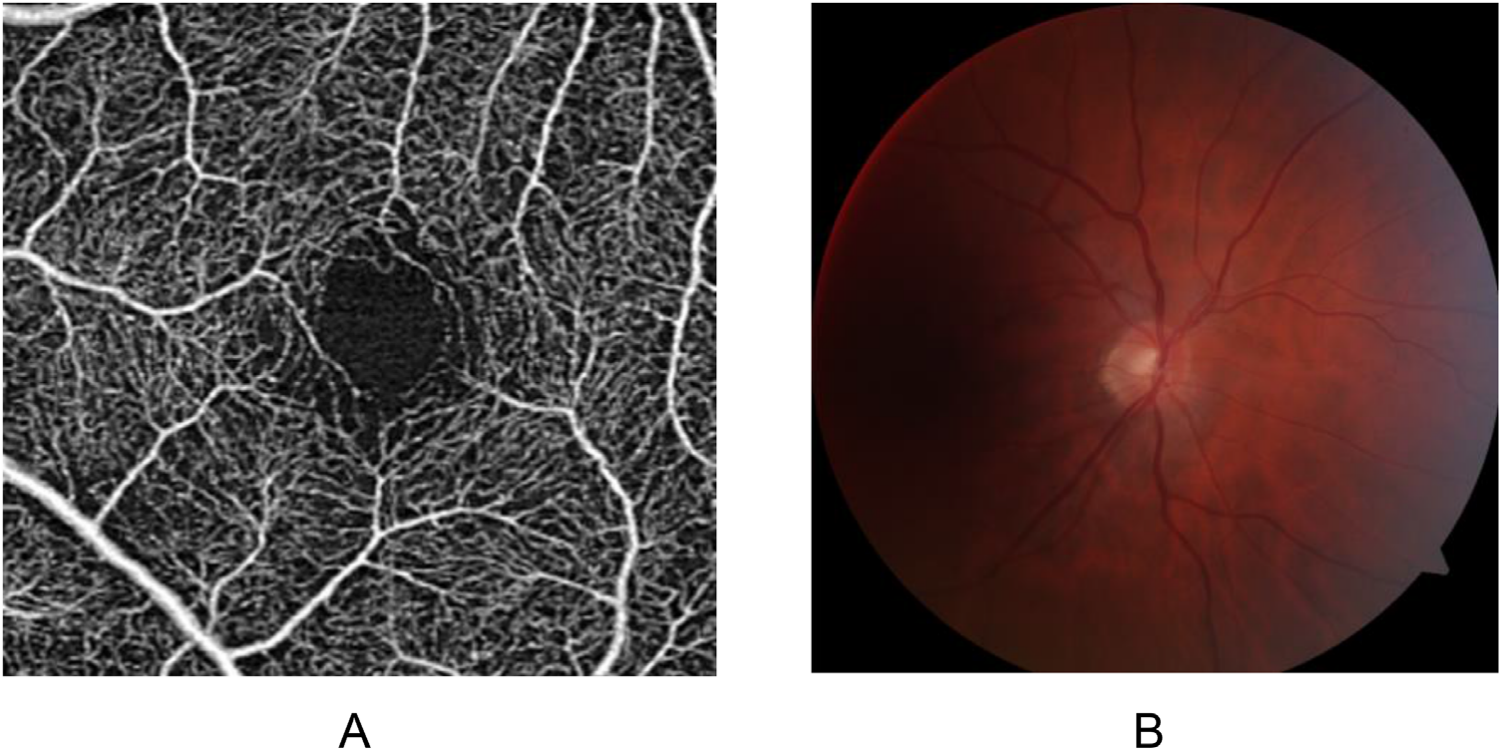
Retinal microvascular image. (**A**) Retinal superficial capillary plexus with optical coherence tomography angiography examinations. (**B**) Forty-five-degree color retinal photographs, centered on the optic disc.

After inclusion, the patients underwent an OCT-A examination (CIRRUS HD-OCT, Model 5000; Carl Zeiss Meditec AG) and 45 degree color retinal photographs, centered on the optic disc, were obtained with a fundus camera (TRC NW6S, Topcon, Tokyo, Japan) for both eyes. This eye examination was performed under mydriasis obtained with eye drops containing tropicamide 0.5% (Thea, Clermont-Ferrand, France). Axial length was measured using an optical biometer (IOL Master; Carl Zeiss Meditec AG, Jena, Germany). Quantitative OCT-A metrics were obtained with the angiography software (Angioplex, version 10; Carl Zeiss Meditec AG; Fig. 1A). We investigated the retinal vascular features in the superficial capillary plexus (SCP) and the following measurements were taken in the 3 × 3 mm angiograms: the area of the foveal avascular zone (FAZ; mm^2^), perfusion density (area, unitless), and vessel density (length, mm^−1^). These densities were both measured in the inner and full Early Treatment Diabetic Retinopathy Study (EDTRS) circle.

In addition, fundus photographs were anonymously sent to the reading center in Yamagata University, Japan (author R.K.), and a single trained grader extracted retinal vessel characteristics with the SIVA software (Fig. 1B). The computerized analysis of the retinal vascular network computerized analysis was based on the analysis of vessels from the center of the optic disc and then to three successive zones corresponding to 0.5 (zone A), 1 (zone B), and 2 (zone C) disc diameter. The six largest arterioles and veins were analyzed.

Thus, only one eye (with both OCT-A and SIVA examinations) was retained for analysis for each participant and its selection followed the criteria described below: (1) fundus photograph and OCT-A of the right eye for participants born in even-numbered years and the left eye for those born in odd-numbered years; (2) in single-eye patients, the functional eye was selected; and (3) when SIVA or OCT-A were uninterpretable on one eye, the other one was retained for analysis. Only OCT-A images with a signal strength >7/10 were retained. For this ancillary study we kept 144 eyes with both OCT-A and SIVA examinations.

### Cardiovascular Data Collection

As described previously, we extracted cardiovascular data from medical records and observation sheets used by the Observatoire des Infarctus de Côte d'Or (RICO).^5^ The following data were collected: age, sex, high blood pressure, diabetes mellitus history, body mass index (BMI), hypercholesterolemia, and current smoking. From the above data, cardiovascular risk scores defined by the AHA (AHA risk score) for a high-risk population were calculated. The AHA risk score includes age, sex, the ethnic origin, the history of arterial hypertension and diabetes, active smoking, systolic, and diastolic arterial pressure and levels of total cholesterol and high-density lipoprotein (HDL) cholesterol levels.^17^ The anatomic Syntax score, a risk stratification score for coronary lesions (length, bifurcation, diffuse disease, calcifications, thrombus, and total occlusion) was determined for all of the patients who underwent coronarography.^18^ We finally calculated the SCORE risk.^19^

### Pre-Processing of OCT-A and SIVA Data

**Figure 2.**
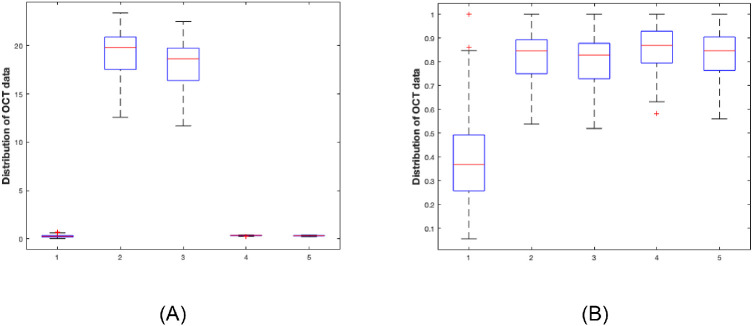
Distribution of optical coherence tomography angiography parameters values 1: Foveal avascular zone; 2: Vessel inner; 3: Vessel full; 4: Perfusion inner; 5: Perfusion full. (**A**) Original data, (**B**) normalized data between 0 and 1.

**Figure 3.**
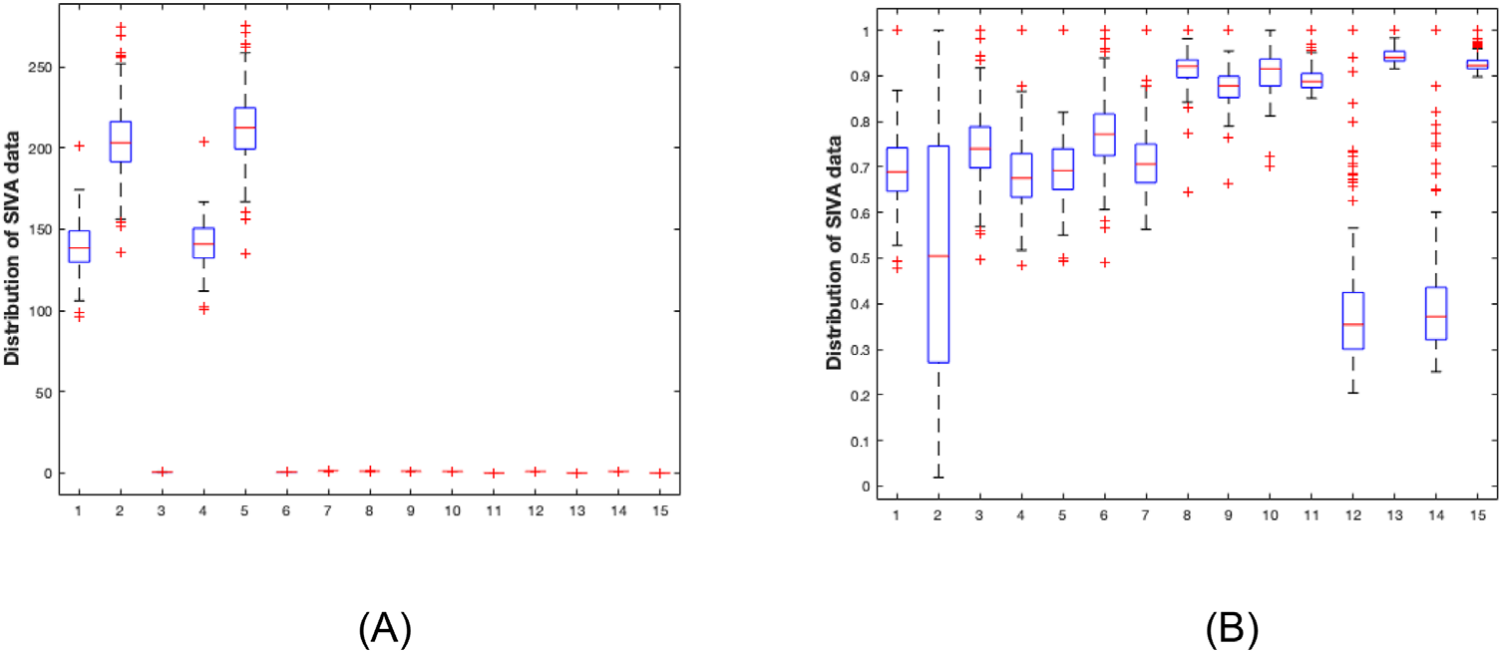
Distribution of Singapore “I” Vessel Assessment parameters values: 1: Biggest six arterioles in Zone B 2: Biggest six veins in Zone B 3: Arteriole - Venular Ratio of Zone B 4: Biggest six arterioles in Zone C 5: Biggest six veins in Zone C 6: Arteriole - Venular Ratio of Zone C 7: Fractal dimension total zone C 8: Fractal dimension arterioles zone C 9: Fractal dimension venules zone C 10: Simple tortuosity arteriole 11: Curvature tortuosity arteriole 12: Simple tortuosity venule 13: Curvature tortuosity venule 14: Simple tortuosity vessels 15: Curvature tortuosity vessels. (**A**) Original data, (**B**) normalized data between 0 and 1.

The dispersion of the value of the different parameter is significant from one patient to another and from one parameter to another. The scale of values also differs. To provide a common referential for all data, these were normalized between 0 and 1. The SIVA and OCT-A settings used in this study are listed in Figures 1 and 2. Figure 2A and Figure 3A illustrate this variability and dispersion of values. Figure 2B and Figure 3B show the normalized data.

### Machine Learning Approach

The method used can be summarized by the following conceptual framework:

1. To build a prediction model of the cardiovascular risk score and parameters based on supervised machine learning approach. The process applied a set of known input (OCT-A and/or SIVA data) with response data (cardiovascular risk score and parameters) and created a model to produce reasonable predictions of these cardiovascular parameters. In this study, supervised learning based on classification (K-nearest neighbors, discriminant analysis, and Naïve Bayes) and regression (decision trees) techniques were used in order to develop these predictive models.
2. To use the predictive model obtained in the previous step to estimate the cardiovascular parameters of patients depending on OCT-A and SIVA retinal vascular features.

Further details on the methodology were provided in the Supplementary Material. The cardiovascular parameters were divided into two groups. The first group consisted of the cardiovascular risk score: AHA Risk score, Syntax score, and SCORE risk. Regarding the AHA Risk score in this study, it has not been used as a primary prevention risk assessment as it was calculated in a high-risk profile population. We chose this score to attest the systemic vascular profile of our population because no therapeutic decision (aspirin, statins, and antihypertensive drugs) was made with regard to this calculation. This group accounted for the primary prediction goal. The second one included age, sex, high blood pressure history, diabetes mellitus history, hypercholesterolemia, current smoking status, and BMI.

In this study, each cardiovascular parameter was categorized using two or three labels (Score risk ≥ 5%, AHA risk ≥ 20%, Score syntax ≥ 33, age > 60 years, and BMI > 25 and > 30). To avoid bias in the choice of the learning group patients, patients were selected randomly. In our algorithm, the minimum number of patients required for the learning group was not defined. The learning group increased in increments of 10 patients. For each increment, the predictive model was applied to the database in order to investigate the correlation between the prediction rate and the size of the learning group. All programs were written using Matlab software. These programs can be shared on request to the authors. For each data set, the prediction rates of the four ML algorithms were compared. After that, the prediction rates of the same algorithm were compared according to the three data sets (OCT versus SIVA versus OCT + SIVA). Paired tests with post hoc correction using Tukey pair wise multiple comparisons were used.^20^ This analysis was done using the mixed procedure of SAS statistical software (version 9.4; SAS Institute, Inc., Cary, NC, USA).

## Results

In this study, the data of 144 patients were taken into account. Baseline characteristics of the study participants are presented in Table 1. The mean age was 61.9 (±12.6) years old and 20.1% were female patients. There was no difference between participants and nonparticipants from the EYE-MI study.^5^ The prediction rates of cardiovascular parameters based on the K-nearest neighbor (KNN) approach and the Naïve Bayes approach are presented in Supplementary Figures S1 and S2. These figures show the value of the prediction rate depending on the number of learning patients.

**Table 1.** Baseline Characteristics Between Participants and Nonparticipants

	Global EYE-MI Population *N* = 237	Participants *N* = 144	Nonparticipants *N* = 93	*P* Value
**Cardiovascular characteristics**				
Age, y	62.0 (±13.0)	61.9 (±12.6)	61.4 (±12.8)	0.643
Gender, female	51.0 (21.5)	29.0 (20.1)	22.0 (23.7)	0.630
Previous high blood pressure	121.0 (51.0)	74.0 (51.4)	47.0 (50.5)	1.0
Previous diabetes	53.0 (22.4)	35.0 (24.3)	18.0 (19.4)	0.463
Active smoking	67.0 (28.3)	40.0 (27.8)	27.0 (29.0)	0.578
Body mass index, m²/kg	26.7 (±5.7)	27.1 (±4.2)	27.3 (±4.7)	0.677
Hypercholesterolemia	96.0 (40.5)	61.0 (42.4)	35.0 (37.6)	0.556
Family history of CHD	80.0 (33.8)	52.0 (36.1)	28.0 (30.1)	0.416
Systolic pressure at admission, mm Hg	144.1 (±29.8)	143.0 (± 29.9)	144.2 (±29.5)	0.864
Diastolic pressure at admission, mm Hg	84.2 (±18.7)	83.6 (±19.8)	84.4 (±17.2)	0.753
LVEF at admission, %	54.0 (±10.7)	53.9 (±11.1)	54.2 (±10.2)	0.835
**Vascular history**				
Ischemic coronary heart disease	51.0 (21.5)	35.0 (24.3)	16.0 (17.2)	0.256
Carotid atheroma	10.0 (4.2)	7.0 (4.9)	3.0 (3.2)	0.779
Peripheral artery disease	12.0 (5.1)	8.0 (5.6)	4.0 (4.3)	0.899
Chronic kidney failure	7.0 (3.0)	6.0 (4.2)	1.0 (1.1)	0.327
Ischemic stroke	9.0 (3.8)	4.0 (2.8)	5.0 (5.4)	0.507
**Acute coronary syndrome**				
STEMI	94.0 (39.7)	54.0 (37.5)	40.0 (43.0)	0.626
NSTEMI	113.0 (47.6)	70.0 (48.6)	43.0 (46.2)	0.645
Unstable angina	30.0 (12.7)	20.0 (13.9)	10.0 (10.8)	0.756
**Cardiovascular risk scores**				
AHA Risk score	19.8 (±14.5)	18.7 (±14.7)	21.5 (±14.1)	0.145
Syntax score	11.6 (±9.5)	11.5 (±9.7)	11.9 (±9.2)	0.759
SCORE risk	3.5 (±2.8)	3.2 (2.3)	2.7 (±1.7)	0.364

The results are displayed as *n* (%) for categorical variables and as mean and standard deviation M (±SD) for continuous variables.

CHD, Cardiovascular and heart disease; LVEF, Left ventricular ejection fraction; NSTEMI, Non ST-Elevation Myocardial Infarction; STEMI, ST-Elevation Myocardial Infarction. *P* value for comparison between participants and non-participants.

**Figure 4.**
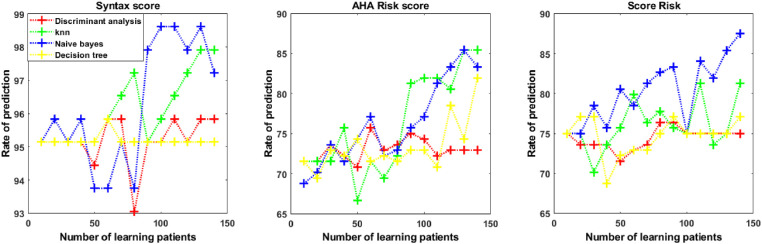
Prediction model for cardiovascular risk score with optical coherence tomography angiography data according to four machine learning algorithms (K-nearest neighbors [KNN], discriminant analysis, Naïve Bayes, and decision tree).

**Figure 5.**
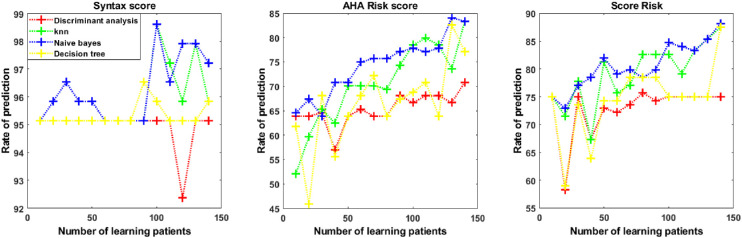
Prediction model for cardiovascular risk score with Singapore “I” Vessel Assessment data according to four machine learning algorithms (K-nearest neighbors [KNN], discriminant analysis, Naïve Bayes, and decision tree).

**Figure 6.**
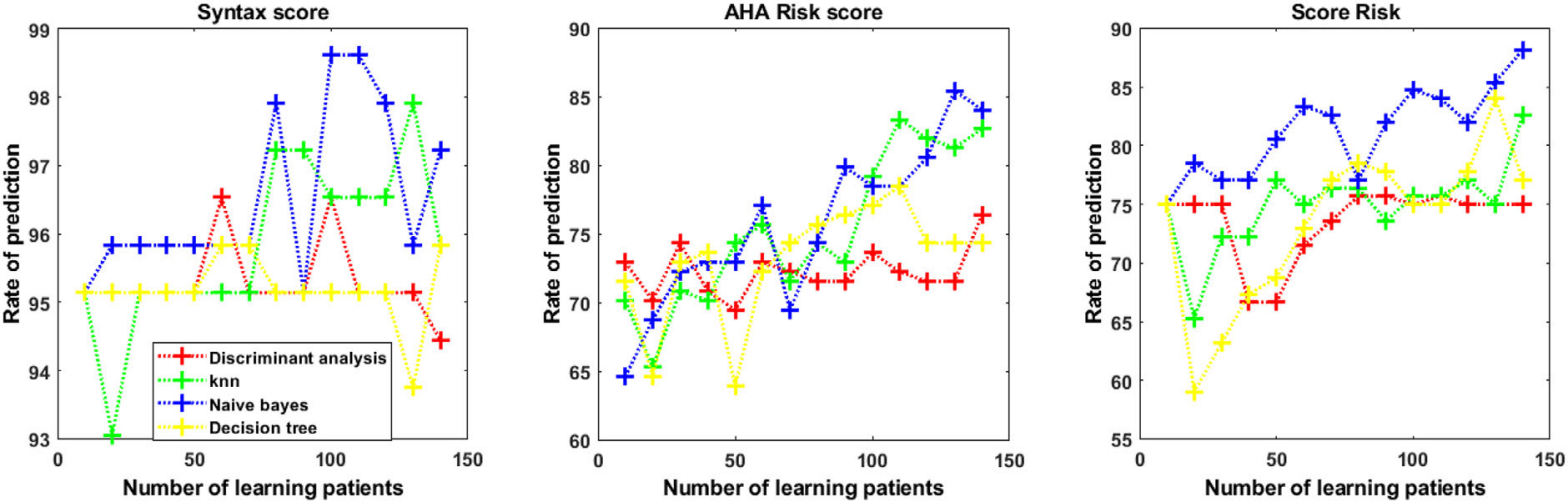
Prediction model for cardiovascular risk score with combined optical coherence tomography angiography + Singapore “I” Vessel Assessment data according to four machine learning algorithms (K-nearest neighbors [KNN], discriminant analysis, Naïve Bayes, and decision tree).

Figures 4, 5, and 6 displayed the comparison of the prediction results from the four ML algorithms for the cardiovascular risk score.

As shown in Figures 4, 5, and 6, the prediction rate was dependent on the number of learning patients used to build the prediction model. The prediction rates of the four ML techniques are reported in Table 2 from OCT-A data, Table 3 from SIVA data, and Table 4 from OCT-A + SIVA data. There were significant differences between prediction rates obtained from the discriminant analysis, the KNN method, the Naïve Bayes approach, and the decision tree classification with the Type 3 Tests of Fixed Effects. When using multiple test adjustment, two algorithms (Naïve Bayes and KNN) were associated with higher prediction rates compared to the discriminant analysis and the decision tree classification (Supplementary Table). The ranges of accuracy were for KNN and OCT-A + SIVA data (0.25–0.97), OCT-A data (0.31–0.98), and SIVA data (0.24–0.98). For the Naïve Bayes approach the range of accuracy were OCT-A + SIVA data (0.24–0.98), OCT-A data (0.29–0.98), and SIVA data (0.39–0.97). For both strategies, sensitivity and specificity ranged from 0 to 1. The KNN and the Naïve Bayes approaches more accurately predicted the three cardiovascular risk scores compared to the discriminant analysis and the decision tree approaches. Finally, we compared the prediction rate with SIVA, OCT-A, and OCT-A + SIVA data for these two algorithms (Naïve Bayes and KNN) and each cardiovascular parameters (Table 5).

**Table 2.** Prediction Rate of the Four Machine Learning Techniques Using the Optical Coherence Tomography Angiography Data (*n* = 144)

	Discriminant Analysis	KNN	Naïve Bayes	Decision Tree	*P* Value
Syntax score	95.19 ± 0.74	96.13 ± 1.08	96.23 ± 1.88	95.19 ± 0.19	0.01
AHA risk score	74.40 ± 1.33	76.09 ± 3.08	80.31 ± 4.14	74.65 ± 2.34	<0.01
SCORE risk	73.02 ± 1.36	76.16 ± 6.33	76.19 ± 5.30	73.36 ± 3.24	0.02
Age	63.05 ± 1.62	70.34 ± 8.55	73.07 ± 8.26	68.60 ± 6.42	<0.01
Sex, male	77.63 ± 5.47	77.43 ± 7.49	77.63 ± 5,47	77.28 ± 8.27	<0.01
High blood pressure history	53.62 ± 3.68	67.56 ± 8.30	69.79 ± 10.19	62.45 ± 7.85	<0.01
Diabetes mellitus history	75.45 ± 2.30	78.08 ± 5.83	75.45 ± 2.30	73.91 ± 5.17	<0.01
Hypercholesterolemia	56.15 ± 3.82	70.44 ± 11.16	66.47 ± 9.06	59.72 ± 6.41	<0.01
Current smoking	46.43 ± 4.55	56.00 ±12.70	59.52 ± 13.06	48.86 ± 10.08	<0.01
Body mass index	40.53 ± 9.32	58.23 ±11.95	58.98 ± 12.97	51.14 ± 10.86	<0.01

The prediction rates (%) are displayed as mean and standard deviation (M ± SD).

**Table 3.** Prediction Rate of the Four Machine Learning Techniques Using the Singapore “I” Vessel Assessment Data (*n* = 144)

	Discriminant Analysis	KNN	Naïve Bayes	Decision Tree	*P* Value
Syntax score	94.94 ± 0.74	95.83 ± 1.19	96.28 ± 1.21	95.34 ± 0.42	<0.01
AHA risk score	72.82 ± 4.68	79.22 ± 5.55	80.61 ± 4.24	74.50 ± 6.62	<0.01
SCORE risk	65.33 ± 3.25	70.54 ± 8.56	74.36 ± 6.17	66.42 ± 8.87	<0.01
Age	54.56 ± 4.65	66.27 ± 10.06	54.56 ± 4.65	63.69 ± 7.32	<0.01
Sex, male	79.66 ± 0.74	79.22 ± 12.86	84.47 ± 6.14	78.77 ± 7.89	0.09
High blood pressure history	59.03 ± 4.73	64.83 ± 10.74	67.71 ± 8.51	66.52 ± 7.08	<0.01
Diabetes mellitus history	74.21 ± 3.62	80.36 ± 5.31	82.29 ± 6.60	72.87 ± 6.27	<0.01
Hypercholesterolemia	60.22 ± 3.90	69.84 ± 9.27	69.94 ± 9.55	63.89 ± 8.85	<0.01
Current smoking	44.94 ± 5.60	54.02 ± 15.92	59.62 ± 16.82	50.79 ± 11.88	<0.01
Body mass index	47.47 ± 5.25	59.87 ± 10.84	60.86 ± 14.48	53.08 ± 9.48	<0.01

The prediction rates (%) are displayed as mean and standard deviation (M ± SD).

**Table 4.** Prediction Rate of the Four Machine Learning Techniques Using the Both Optical Coherence Tomography Angiography and the Singapore “I” Vessel Assessment Data (*n* = 144)

	Discriminant Analysis	KNN	Naïve Bayes	Decision Tree	*P* Value
Syntax score	95.14 ± 0.82	95.83 ± 1.25	96.53 ± 1.25	95.19 ± 0.51	<0.01
AHA risk score	73.61 ± 3.13	74.95 ± 3.76	81.25 ± 3.84	73.46 ± 6.68	<0.01
SCORE risk	72.22 ± 1.77	75.25 ± 5.61	75.64 ± 5.96	73.12 ± 4.20	0.03
Age	61.36 ± 6.49	70.63 ± 6.95	70.14 ± 10.55	69.20 ± 7.46	<0.01
Sex, male	77.83 ± 6.87	77.73 ± 11.59	86.46 ± 5.08	78.37 ± 8.83	<0.01
High blood pressure history	60.37 ± 4.91	67.46 ± 10.94	66.96 ± 9.63	67.81 ± 7.97	<0.01
Diabetes mellitus history	76.24 ± 3.36	79.86 ± 4.58	83.48 ± 5.50	75.10 ± 5.71	<0.01
Hypercholesterolemia	61.61 ± 5.09	71.03 ± 12.16	70.09 ± 9.82	66.12 ± 8.68	<0.01
Current smoking	48.16 ± 6.58	58.28 ± 13.67	59.97 ± 15.54	50.94 ± 12.27	<0.01
Body mass index	47.82 ± 4.65	59.57 ± 12.28	60.37 ± 14.67	51.89 ± 10.12	<0.01

The prediction rates (%) are displayed as mean and standard deviation (M ± SD).

**Table 5.** Comparison of Prediction of Cardiovascular Parameters With Machine Learning With OCT-A, SIVA, and OCT-A + SIVA

	Reference	Compared Strategy	Estimate	Standard Error	Adjusted *P* Value
**Naïve Bayes**					
Age					
	OCT-A	OCT-A + SIVA	2.93	1.48	0.14
	SIVA	OCT-A + SIVA	−2.08	1.48	0.35
High blood pressure history					
	OCT-A	OCT-A + SIVA	2.83	0.69	<0.01
	SIVA	OCT-A + SIVA	0.74	0.69	0.53
Hypercholesterolemia					
	OCT-A	OCT-A + SIVA	−3.62	1.21	0.02
	SIVA	OCT-A + SIVA	−0.15	1.21	0.99
**KNN**					
AHA risk score					
	OCT-A	OCT-A + SIVA	1.14	1.19	0.61
	SIVA	OCT-A + SIVA	4.27	1.19	<0.01
SCORE risk					
	OCT-A	OCT-A + SIVA	0.94	1.40	0.78
	SIVA	OCT-A + SIVA	−4.71	1.40	<0.01
Age					
	OCT-A	OCT-A + SIVA	−0.2976	1.5351	0.98
	SIVA	OCT-A + SIVA	−4.3651	1.5351	0.02

OCT-A, optical coherence tomography angiography; SIVA, the Singapore “I” Vessel Assessment.

## Discussion

In this cross-sectional pilot study, we applied a prediction model of cardiovascular parameters from ML algorithms using retinal vascular characteristics measured with SIVA software and OCT-A (alone and combined). Overall, we observed that this approach may be effective to procure a moderate to robust prediction rate for a specific cardiovascular data set.

To the best of our knowledge, this is the first study to explore the potential interest of using quantitative parameters of the retinal microvascular network measured by means of SIVA software and OCT-A to predict the cardiovascular risk factors burden of high cardiovascular risk profile patients with a non-deep learning model. In this study, we focused on patients with a history of cardiovascular disease (inclusion criteria: ACS). OCT-A and retinal vascular analysis based on fundus photographs with software, such as SIVA, provide us with a large amount of quantitative data. Supervised classifiers help us to properly take advantage of all these quantitative data. Although deep learning based on convolutional neural networks in ophthalmology is commonly used to detect glaucoma, aged-related macular degeneration and diabetic retinopathy,^21^^–^^24^ the sparsity of OCT-A and SIVA data added to our relatively small sample size make it difficult to apply this type of deep learning algorithm in this study.^25^^,^^26^ In this regard, our approach of cardiovascular prediction with retinal vascular biomarker is novel and helpful.

Publications concerning the prediction of the cardiovascular risk profile with ML algorithm are steadily increasing in number.^27^^,^^28^ Alaa et al. recently investigated the cardiovascular disease risk prediction with ML methods.^29^ They conducted a large prospective cohort study and analyzed data on 423,604 participants without cardiovascular disease at baseline in UK Biobank. They used more than 470 variables, notably on health and medical history, dietary and nutritional information, and sociodemographics. They found that their ML algorithm significantly improved the accuracy of cardiovascular disease risk prediction compared to gold standard scoring systems based on conventional risk factors (Framingham score).^30^

Comparison with Poplin et al.'s results (prediction for age only area under the concentration curve [AUC] = 0.66 [0.61 to 0.71] and for the risk SCORE AUC = 0.72 [0.67 to 0.76]) is impossible because our strategy of using the quantitative dataset and the supervised ML model is significantly different.^12^ The UK Biobank provides a tremendous amount of information in a very large population, making it possible to predict cardiovascular parameters and more recently hemoglobin concentration.^31^ Moreover, Gerrits et al. recently published a deep learning algorithm to predict cardiometabolic risk factors based on 12,000 retinal images from 3000 participants.^32^ Recently, Cheung et al. suggested creating a “retinal vessel CVD risk score” based on the artificial intelligence system.^33^ This deep learning project will have to overcome differences in terms of imaging device, image quality, and protocol standardization. Our approach is more pragmatic and could be reserved for smaller centers with a limited data set. Furthermore, in our algorithms, we were able to include different kinds of retinal image parameters: SIVA and OCT-A combined and not only retinal vessel caliber.

In this study, prediction rates were moderate to high for all the cardiovascular risk factors, which was particularly true for diabetic status, the Syntax risk score, AHA risk score, and SCORE risk score. Even if patients with diabetes did not present with diabetic retinopathy, the prediction rates were high with the four algorithms for SIVA, OCT-A alone, and SIVA and OCT-A combined. These findings demonstrate that vascular changes are significant before incidence of diabetic retinopathy.^34^^–^^36^ The present study is also valuable because of the good prediction rate not only for cardiovascular risk factors but also for well-established cardiovascular scores with Naïve Bayes and SIVA + OCT-A data: the AHA risk score 81.25%, SCORE risk 75.64%, and 96.53% for the Syntax score.

We compared the four algorithms with multiple test adjustments. Overall, Naïve Bayes and KNN gave a higher prediction rate compared to the discriminant analysis and the decision tree classification for every cardiovascular parameter. Comparison of SIVA, OCT-A alone, or combined did not demonstrate clear superiority of one technique over the others. Very different retinal vascular characteristics could be extracted from OCT-A and SIVA. With OCT-A, one could extract the fovea and capillary network features, whereas with SIVA from fundus photographs represent the analysis of venules and arterioles from the center of the optic disc and then to three successive peripheral zones. We aimed to combine both in order to give a thorough description of the retinal vascular network as a whole. Interestingly, SIVA and OCT-A combined did not give better predictive rate compared to SIVA or OCT-A alone. We could hypothesize that predictive vascular information is already high with one single device and that extra retinal quantitative data are not incremental. Multimodal imaging could be useful in future studies to predict continuous cardiovascular parameters and not with label (two or three in this study).

At the moment, machine and deep learning algorithms are more focused on fundus photographs.^10^^,^^37^ We showed that applying ML Bayesian classifier on OCT-A quantitative data about retinal microvascularization (solo or combined) could also be of interest in predictive models.

We acknowledge several limitations to this study. First, one should remain very cautious regarding these findings given the small size of the input data set (144 patients, 5 retinal vascular parameters on OCT-A and 15 parameters with SIVA software). Second, the robust prediction rates could be related to the definition of two or three labels for each cardiovascular parameter. Third, we only considered one eye per patient. This selection was established on the basis of the quality of images creating a selection bias within each patient. Furthermore, if retinal quantitative parameters had been available for both eyes, we could have used one eye from the participant to train the model and used the other eye for validation. In future studies, we should consider each eye individually. Fourth, very high image acquisition quality is mandatory for this type of study. The predictive model performance could be impaired by the technical limitations of each imaging device (artifacts, segmentation abnormalities, and signal strength). Jammal et al. proposed a deep learning algorithm to detect segmentation errors on OCT scans for retinal nerve fiber layer measurement.^38^ In future studies, this kind of approach could help us to improve the quality of the images’ data set. Fifth, this was a cross-sectional study. Future cardiovascular events were unknown. As a consequence, we were not able to evaluate the incremental value of the ML predictive model based on retinal parameters compared to the usual risk score. Sixth, regarding OCT-A parameters, we only collected foveal vasculature structure (foveal avascular zone, vessel inner and full, and perfusion inner and full) by means of the Angioplex software (version 10; Carl Zeiss Meditec AG). Additional OCT-A retinal biomarkers, such as fractal dimension, could in the future improve our prediction rates. Seventh, we used the same data set for training the algorithms and for testing their prediction accuracy. In future studies, we could use an external dataset to improve our accuracy. Finally, our algorithm was applied to a population with a high cardiovascular risk profile. At the moment, it could not be replicated in a healthy population for primary prevention. Furthermore, a comparison group of healthy patients could help in future longitudinal studies to improve the algorithms’ performance.

In conclusion, these preliminary findings demonstrate that ML algorithms applied to quantitative retinal vascular parameters with SIVA software and OCT-A show a good predictive performance of cardiovascular risk factors and cardiovascular risk scores. Quantitative retinal vascular biomarkers combined with an artificial intelligence strategy might be valuable data to implement a predictive model for cardiovascular parameters.

## Supplementary Material

Supplement 1

Supplement 2

Supplement 3
