# Supplementary material for: Prediction of Cardiovascular Parameters With Supervised Machine Learning From Singapore “I” Vessel Assessment and OCT-Angiography: A Pilot Study
Source: Transl Vis Sci Technol. 2021 Nov 12;10(13):20. doi: 10.1167/tvst.10.13.20 (PMC8590163; doi:10.1167/tvst.10.13.20)
Supplement: Supplement 1 [file tvst-10-13-20_s001.docx]

Supplementary material: Machine Learning methodology

We based our methodology on the following three steps :

1. During the first step, data and labels are divided in the training group and in the test group.

Training data (OCT, SIVA)

Training labels

(cardiovascular features)

Test data (OCT, SIVA)

Test labels

(cardiovascular features)

Data (OCT, SIVA)

Labels

(cardiovascular features)

The imbalance of the number of patients between the classes (labels) introduces a bias in the training or learning process and also in the prediction results. The different classes of labels of the cardiovascular features is not fairly distributed as you can see in the Figure 1.


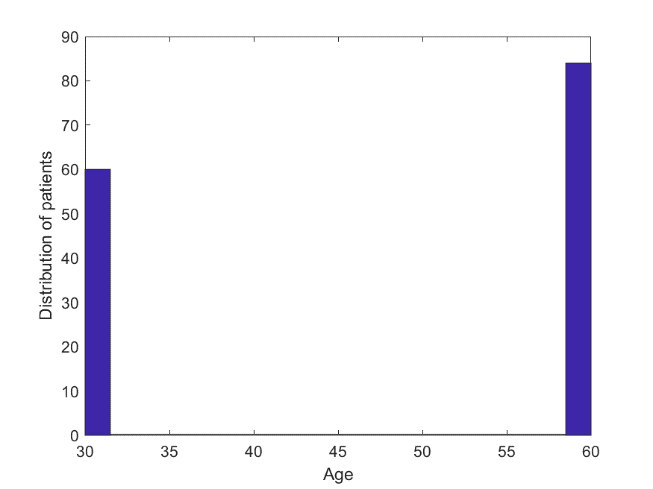

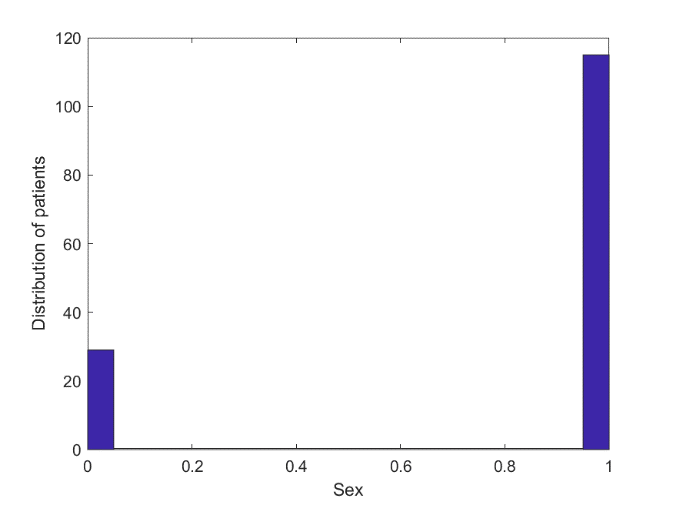


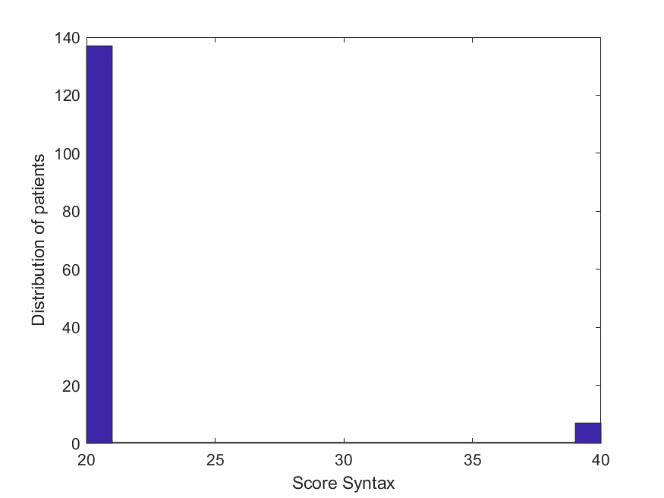

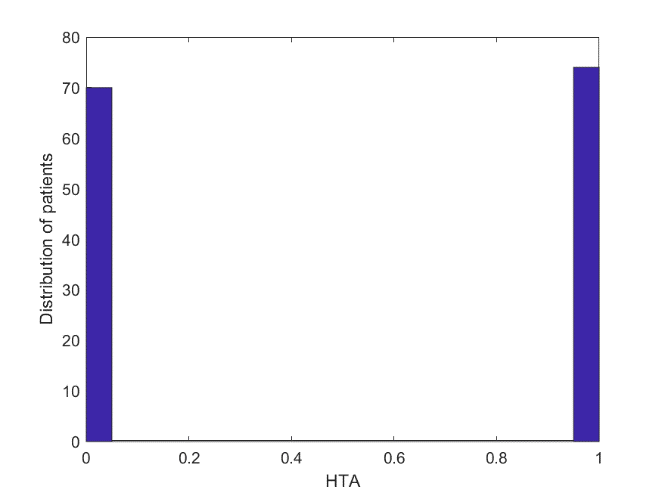


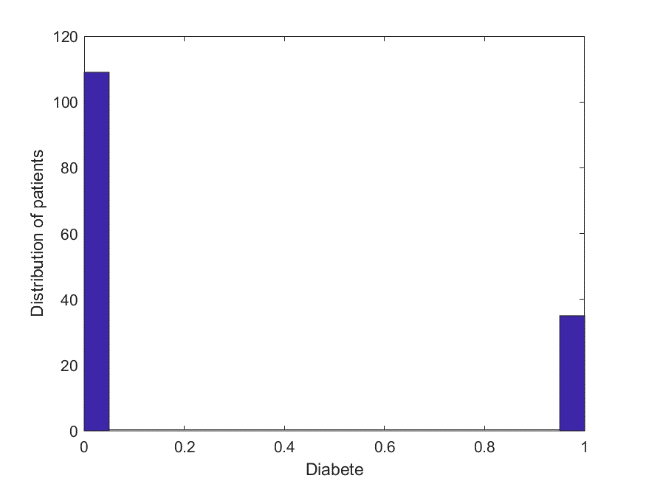

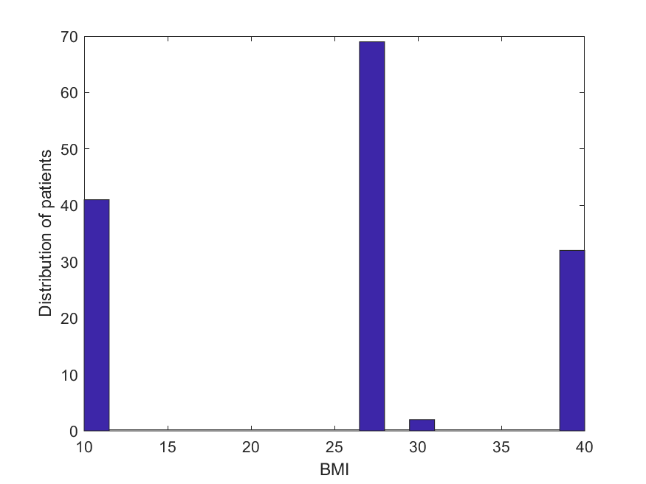


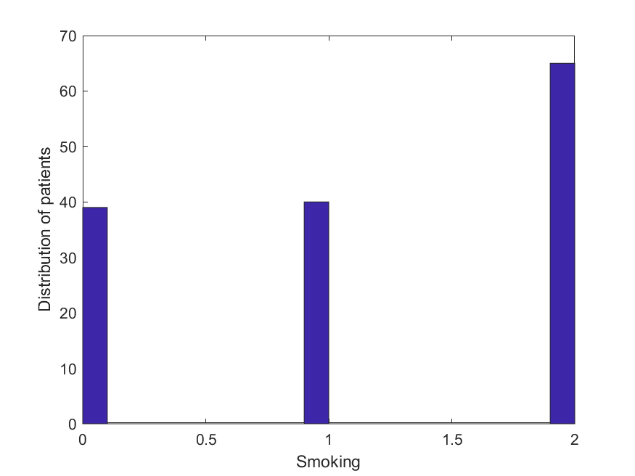

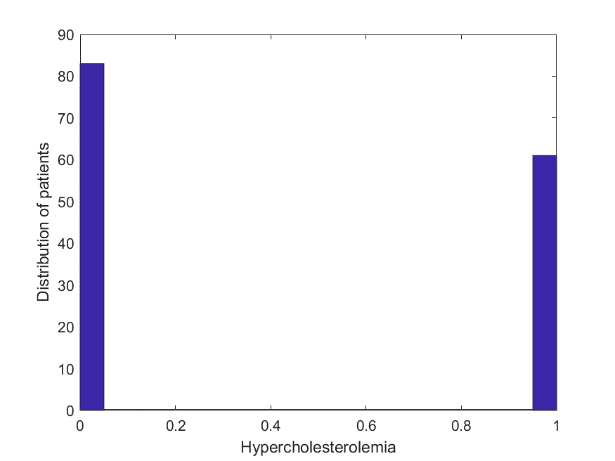


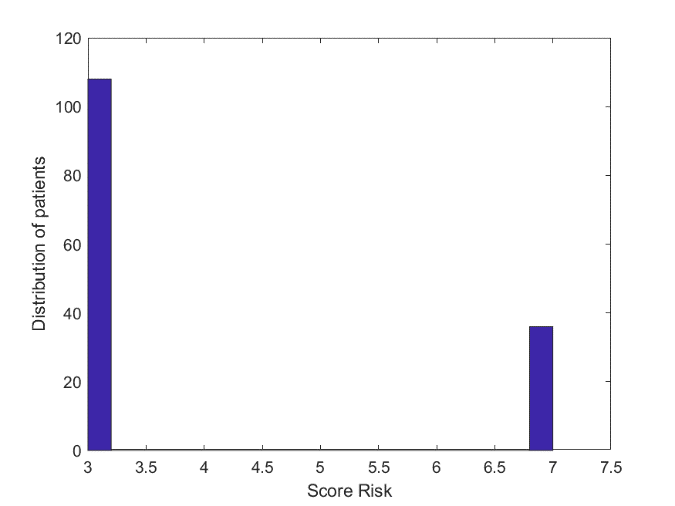

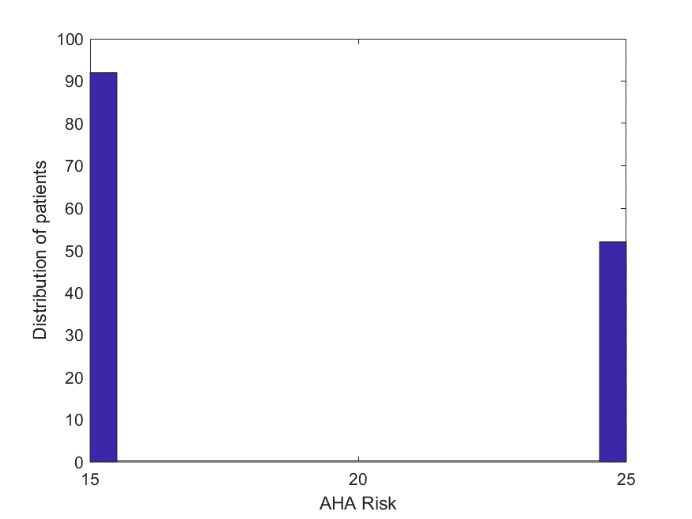


Figure 1 : Distribution of the patient data on the different labels of the cardiovascular features

Considering this distribution of the data, a random process has been used to define the training data and to construct the prediction model. This is not an optimal way but it is a first approach in order to show the influence of the choice of the training data.

Example of the process in order to build the ML model

Original data

| P1 | P2 | P3 | P4 | P5 | P6 | P7 | P8 | P9 | P10 |
| --- | --- | --- | --- | --- | --- | --- | --- | --- | --- |

Random choice of learning or traning data

1st case : 3 training patients and 7 test patients

| P1 | P2 | P3 | P4 | P5 | P6 | P7 | P8 | P9 | P10 |
| --- | --- | --- | --- | --- | --- | --- | --- | --- | --- |

test data

learning or training data

2nd case : 5 training patients and 5 test patients

| P1 | P2 | P3 | P4 | P5 | P6 | P7 | P8 | P9 | P10 |
| --- | --- | --- | --- | --- | --- | --- | --- | --- | --- |

test data

learning or training data

3rd case : 7 training patients and 3 test patients

| P1 | P2 | P3 | P4 | P5 | P6 | P7 | P8 | P9 | P10 |
| --- | --- | --- | --- | --- | --- | --- | --- | --- | --- |

test data

learning or training data

1. During the second step, based on the training group, a prediction model is built using supervised learning based on classification (K-nearest neighbors, discriminant analysis, Naïve Bayes) and regression (decision trees) techniques.

Training data

Training labels

Learning algorithm

Model

Hyperparameter values

1. During the last step, the model of prediction is applied on the test group and the performance of the model is computed.

Test data

Prediction

Performance

Test labels

Model
