# Supplementary material for: Prediction of Cardiovascular Parameters With Supervised Machine Learning From Singapore “I” Vessel Assessment and OCT-Angiography: A Pilot Study
Source: Transl Vis Sci Technol. 2021 Nov 12;10(13):20. doi: 10.1167/tvst.10.13.20 (PMC8590163; doi:10.1167/tvst.10.13.20)
Supplement: Supplement 2 [file tvst-10-13-20_s002.docx]

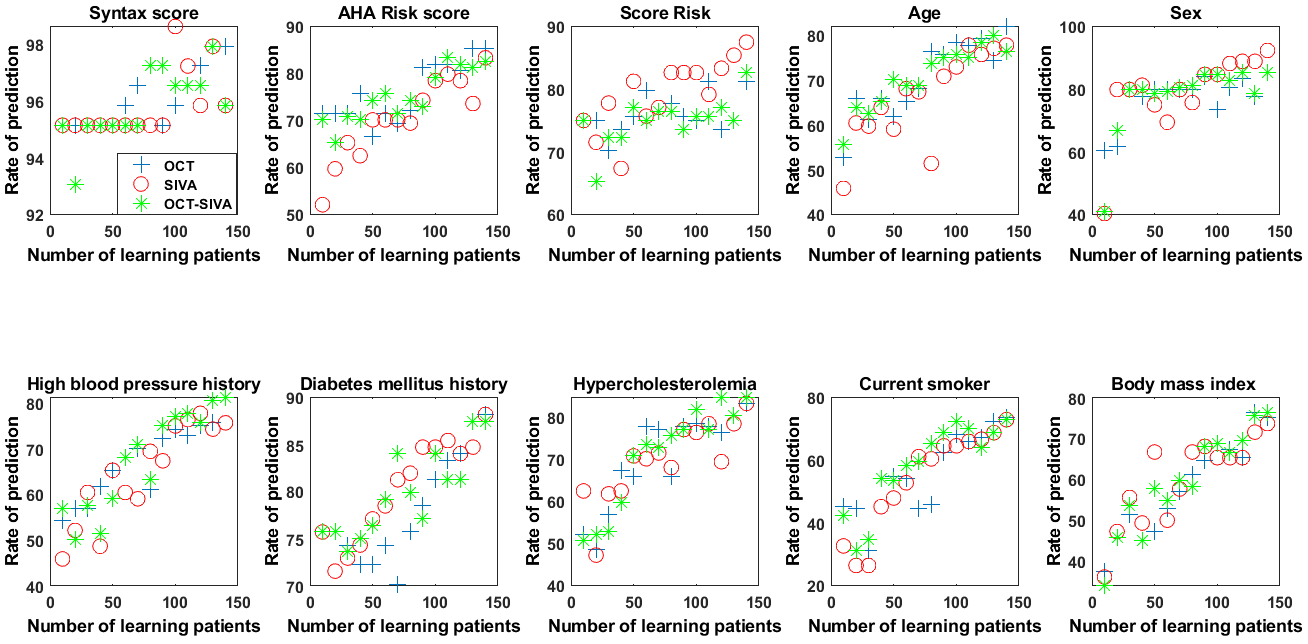


Supplementary Figure S1. Prediction results of cardiovascular parameters using a model based on the KNN approach (OCT-A parameters: blue cross, SIVA parameters: red circle, set of OCT-A + SIVA parameters: green star)

**
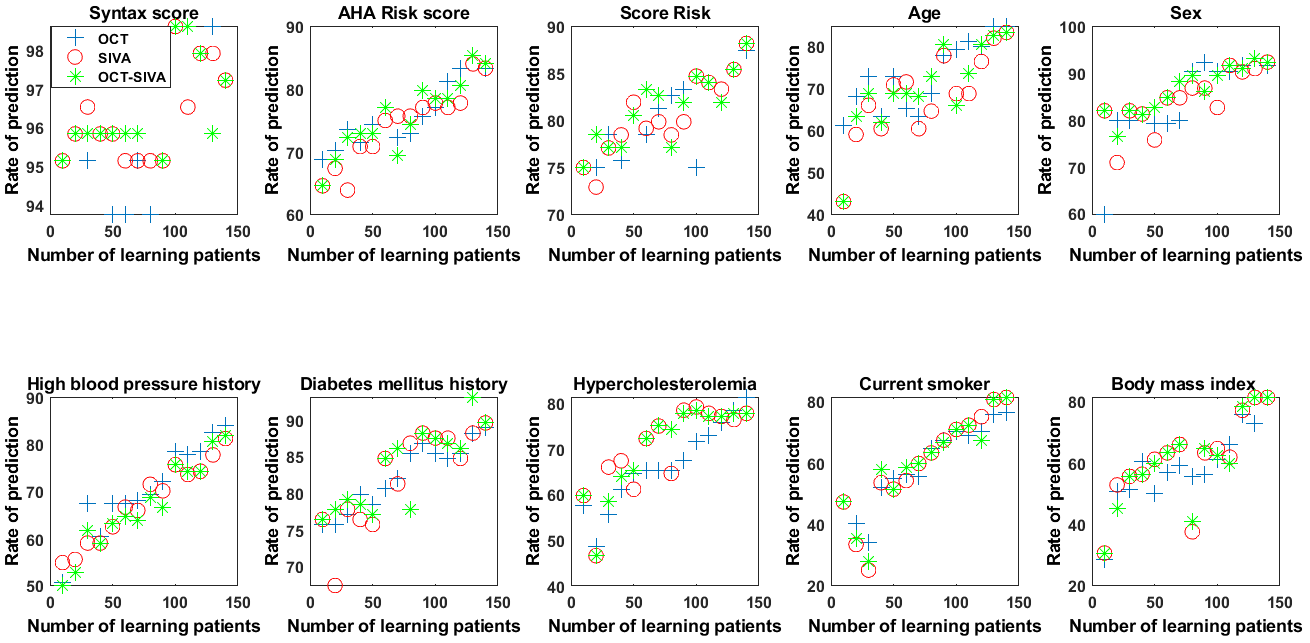
**

Supplementary Figure S2. Prediction results of cardiovascular parameters using a model based on the Naïve Bayes approach (OCT-A parameters: blue cross, SIVA parameters: red circle, set of OCT-A + SIVA parameters: green star)
