# Supplementary material for: Prediction of Cardiovascular Parameters With Supervised Machine Learning From Singapore “I” Vessel Assessment and OCT-Angiography: A Pilot Study
Source: Transl Vis Sci Technol. 2021 Nov 12;10(13):20. doi: 10.1167/tvst.10.13.20 (PMC8590163; doi:10.1167/tvst.10.13.20)
Supplement: Supplement 3 [file tvst-10-13-20_s003.docx]

| OCT-A data |  |  |  | **Estimate** | **Adjusted P-value** |
| --- | --- | --- | --- | --- | --- |
|  | AHA risk score |  |  |  |  |
|  |  | Decision tree | Discriminant analysis | 0.2479 | 0.9935 |
|  |  | Decision tree | KNN | -1.4385 | 0.4312 |
|  |  | Decision tree | Naïve Bayes | -5.6548 | <.0001 |
|  |  | Discriminant analysis | KNN | -1.6864 | 0.2929 |
|  |  | Discriminant analysis | Naïve Bayes | -5.9027 | <.0001 |
|  |  | KNN | Naïve Bayes | -4.2163 | 0.0004 |
|  | Age |  |  |  |  |
|  |  | Decision tree | Discriminant analysis | 5.5554 | 0.0287 |
|  |  | Decision tree | KNN | -1.7362 | 0.7990 |
|  |  | Decision tree | Naïve Bayes | -4.4644 | 0.1057 |
|  |  | Discriminant analysis | KNN | -7.2916 | 0.0025 |
|  |  | Discriminant analysis | Naïve Bayes | -10.0198 | <.0001 |
|  |  | KNN | Naïve Bayes | -2.7281 | 0.4877 |
|  | Sex |  |  |  |  |
|  |  | Decision tree | Discriminant analysis | -0.3471 | 0.9967 |
|  |  | Decision tree | KNN | -0.1488 | 0.9997 |
|  |  | Decision tree | Naïve Bayes | -6.8949 | 0.0009 |
|  |  | Discriminant analysis | KNN | 0.1983 | 0.9994 |
|  |  | Discriminant analysis | Naïve Bayes | -6.5478 | 0.0016 |
|  |  | KNN | Naïve Bayes | -6.7461 | 0.0012 |
|  | High blood pressure history |  |  |  |  |
|  |  | Decision tree | Discriminant analysis | 8.8292 | <.0001 |
|  |  | Decision tree | KNN | -5.1093 | 0.0217 |
|  |  | Decision tree | Naïve Bayes | -7.3414 | 0.0005 |
|  |  | Discriminant analysis | KNN | -13.9385 | <.0001 |
|  |  | Discriminant analysis | Naïve Bayes | -16.1706 | <.0001 |
|  |  | KNN | Naïve Bayes | -2.2321 | 0.5544 |
|  | Diabetes mellitus history |  |  |  |  |
|  |  | Decision tree | Discriminant analysis | -1.5377 | 0.6025 |
|  |  | Decision tree | KNN | -4.1667 | 0.0088 |
|  |  | Decision tree | Naïve Bayes | -8.5317 | <.0001 |
|  |  | Discriminant analysis | KNN | -2.6290 | 0.1620 |
|  |  | Discriminant analysis | Naïve Bayes | -6.9940 | <.0001 |
|  |  | KNN | Naïve Bayes | -4.3651 | 0.0057 |
|  | Hypercholesterolemia |  |  |  |  |
|  |  | Decision tree | Discriminant analysis | 3.5715 | 0.2290 |
|  |  | Decision tree | KNN | -10.7141 | <.0001 |
|  |  | Decision tree | Naïve Bayes | -6.7460 | 0.0040 |
|  |  | Discriminant analysis | KNN | -14.2856 | <.0001 |
|  |  | Discriminant analysis | Naïve Bayes | -10.3175 | <.0001 |
|  |  | KNN | Naïve Bayes | 3.9681 | 0.1544 |
|  | Current smoker |  |  |  |  |
|  |  | Decision tree | Discriminant analysis | 2.4306 | 0.7264 |
|  |  | Decision tree | KNN | -7.1429 | 0.0200 |
|  |  | Decision tree | Naïve Bayes | -10.6647 | 0.0003 |
|  |  | Discriminant analysis | KNN | -9.5734 | 0.0011 |
|  |  | Discriminant analysis | Naïve Bayes | -13.0953 | <.0001 |
|  |  | KNN | Naïve Bayes | -3.5219 | 0.4421 |
|  | Body Mass Index |  |  |  |  |
|  |  | Decision tree | Discriminant analysis | 10.6153 | 0.0035 |
|  |  | Decision tree | KNN | -7.0932 | 0.0799 |
|  |  | Decision tree | Naïve Bayes | -7.8373 | 0.0444 |
|  |  | Discriminant analysis | KNN | -17.7085 | <.0001 |
|  |  | Discriminant analysis | Naïve Bayes | -18.4526 | <.0001 |
|  |  | KNN | Naïve Bayes | -0.7441 | 0.9938 |

Supplementary Table. Multiple test adjustment comparing four algorithms (Naïve Bayes and KNN discriminant analysis and decision tree) with OCT-A and SIVA data

| SIVA data |  |  |  |  |  |
| --- | --- | --- | --- | --- | --- |
|  | Score syntax |  |  |  |  |
|  |  | Decision tree | Discriminant analysis | 0.3968 | 0.6165 |
|  |  | Decision tree | KNN | -0.4960 | 0.4309 |
|  |  | Decision tree | Naïve Bayes | -0.9424 | 0.0295 |
|  |  | Discriminant analysis | KNN | -0.8928 | 0.0427 |
|  |  | Discriminant analysis | Naïve Bayes | -1.3392 | 0.0010 |
|  |  | KNN | Naïve Bayes | -0.4464 | 0.5219 |
|  | AHA risk score |  |  |  |  |
|  |  | Decision tree | Discriminant analysis | 1.6866 | 0.4430 |
|  |  | Decision tree | KNN | -4.7123 | 0.0008 |
|  |  | Decision tree | Naïve Bayes | -6.1011 | <.0001 |
|  |  | Discriminant analysis | KNN | -6.3989 | <.0001 |
|  |  | Discriminant analysis | Naïve Bayes | -7.7876 | <.0001 |
|  |  | KNN | Naïve Bayes | -1.3888 | 0.6050 |
|  | SCORE risk |  |  |  |  |
|  |  | Decision tree | Discriminant analysis | 1.0912 | 0.9194 |
|  |  | Decision tree | KNN | -4.1169 | 0.0932 |
|  |  | Decision tree | Naïve Bayes | -7.9363 | 0.0002 |
|  |  | Discriminant analysis | KNN | -5.2081 | 0.0210 |
|  |  | Discriminant analysis | Naïve Bayes | -9.0275 | <.0001 |
|  |  | KNN | Naïve Bayes | -3.8194 | 0.1332 |
|  | Age |  |  |  |  |
|  |  | Decision tree | Discriminant analysis | 9.1270 | 0.0003 |
|  |  | Decision tree | KNN | -2.5794 | 0.5718 |
|  |  | Decision tree | Naïve Bayes | -4.3651 | 0.1437 |
|  |  | Discriminant analysis | KNN | -11.7064 | <.0001 |
|  |  | Discriminant analysis | Naïve Bayes | -13.4921 | <.0001 |
|  |  | KNN | Naïve Bayes | -1.7857 | 0.8068 |
|  | High blood pressure history |  |  |  |  |
|  |  | Decision tree | Discriminant analysis | 7.4902 | 0.0002 |
|  |  | Decision tree | KNN | 1.6866 | 0.7188 |
|  |  | Decision tree | Naïve Bayes | -1.1905 | 0.8785 |
|  |  | Discriminant analysis | KNN | -5.8036 | 0.0044 |
|  |  | Discriminant analysis | Naïve Bayes | -8.6807 | <.0001 |
|  |  | KNN | Naïve Bayes | -2.8771 | 0.2893 |
|  | Diabetes Mellitus history |  |  |  |  |
|  |  | Decision tree | Discriminant analysis | -1.3393 | 0.7884 |
|  |  | Decision tree | KNN | -7.4901 | <.0001 |
|  |  | Decision tree | Naïve Bayes | -9.4246 | <.0001 |
|  |  | Discriminant analysis | KNN | -6.1508 | 0.0007 |
|  |  | Discriminant analysis | Naïve Bayes | -8.0853 | <.0001 |
|  |  | KNN | Naïve Bayes | -1.9345 | 0.5406 |
|  | Hypercholesterolemia |  |  |  |  |
|  |  | Decision tree | Discriminant analysis | 3.6706 | 3.6706 |
|  |  | Decision tree | KNN | -5.9522 | -5.9522 |
|  |  | Decision tree | Naïve Bayes | -6.0514 | -6.0514 |
|  |  | Discriminant analysis | KNN | -9.6229 | -9.6229 |
|  |  | Discriminant analysis | Naïve Bayes | -9.7221 | -9.7221 |
|  |  | KNN | Naïve Bayes | -0.09921 | -0.09921 |
|  | Current smoker |  |  |  |  |
|  |  | Decision tree | Discriminant analysis | 5.8530 | 0.0970 |
|  |  | Decision tree | KNN | -3.2244 | 0.5602 |
|  |  | Decision tree | Naïve Bayes | -8.8294 | 0.0048 |
|  |  | Discriminant analysis | KNN | -9.0774 | 0.0036 |
|  |  | Discriminant analysis | Naïve Bayes | -14.6824 | <.0001 |
|  |  | KNN | Naïve Bayes | -5.6050 | 0.1196 |
|  | Body Mass Index |  |  |  |  |
|  |  | Decision tree | Discriminant analysis | 5.6051 | 0.1902 |
|  |  | Decision tree | KNN | -6.7958 | 0.0796 |
|  |  | Decision tree | Naïve Bayes | -7.7876 | 0.0347 |
|  |  | Discriminant analysis | KNN | -12.4009 | 0.0003 |
|  |  | Discriminant analysis | Naïve Bayes | -13.3927 | 0.0001 |
|  |  | KNN | Naïve Bayes | -0.9919 | 0.9836 |

| OCT-A + SIVA data |  |  |  |  |  |
| --- | --- | --- | --- | --- | --- |
|  | Score syntax |  |  |  |  |
|  |  | Decision tree | Discriminant analysis | 0.04950 | 0.9989 |
|  |  | Decision tree | KNN | -0.6449 | 0.2596 |
|  |  | Decision tree | Naïve Bayes | -1.3391 | 0.0022 |
|  |  | Discriminant analysis | KNN | -0.6944 | 0.2025 |
|  |  | Discriminant analysis | Naïve Bayes | -1.3886 | 0.0014 |
|  |  | KNN | Naïve Bayes | -0.6942 | 0.2027 |
|  | AHA risk score |  |  |  |  |
|  |  | Decision tree | Discriminant analysis | -0.1488 | 0.9995 |
|  |  | Decision tree | KNN | -1.4880 | 0.6931 |
|  |  | Decision tree | Naïve Bayes | -7.7876 | <.0001 |
|  |  | Discriminant analysis | KNN | -1.3392 | 0.7572 |
|  |  | Discriminant analysis | Naïve Bayes | -7.6388 | <.0001 |
|  |  | KNN | Naïve Bayes | -6.2996 | 0.0002 |
|  | Age |  |  |  |  |
|  |  | Decision tree | Discriminant analysis | 7.8373 | <.0001 |
|  |  | Decision tree | KNN | -1.4386 | 0.7406 |
|  |  | Decision tree | Naïve Bayes | -0.9426 | 0.9091 |
|  |  | Discriminant analysis | KNN | -9.2759 | <.0001 |
|  |  | Discriminant analysis | Naïve Bayes | -8.7799 | <.0001 |
|  |  | KNN | Naïve Bayes | 0.4959 | 0.9850 |
|  | Sex |  |  |  |  |
|  |  | Decision tree | Discriminant analysis | 0.5457 | 0.9918 |
|  |  | Decision tree | KNN | 0.6448 | 0.9867 |
|  |  | Decision tree | Naïve Bayes | -8.0852 | 0.0008 |
|  |  | Discriminant analysis | KNN | 0.09907 | 0.9999 |
|  |  | Discriminant analysis | Naïve Bayes | -8.6309 | 0.0003 |
|  |  | KNN | Naïve Bayes | -8.7300 | 0.0003 |
|  | High blood pressure history |  |  |  |  |
|  |  | Decision tree | Discriminant analysis | 7.4406 | 0.0004 |
|  |  | Decision tree | KNN | 0.3472 | 0.9968 |
|  |  | Decision tree | Naïve Bayes | 0.8431 | 0.9573 |
|  |  | Discriminant analysis | KNN | -7.0934 | 0.0007 |
|  |  | Discriminant analysis | Naïve Bayes | -6.5974 | 0.0017 |
|  |  | KNN | Naïve Bayes | 0.4959 | 0.9907 |
|  | Diabetes Mellitus history |  |  |  |  |
|  |  | Decision tree | Discriminant analysis | -1.1409 | 0.7838 |
|  |  | Decision tree | KNN | -4.7619 | 0.0019 |
|  |  | Decision tree | Naïve Bayes | -8.3829 | <.0001 |
|  |  | Discriminant analysis | KNN | -3.6210 | 0.0243 |
|  |  | Discriminant analysis | Naïve Bayes | -7.2421 | <.0001 |
|  |  | KNN | Naïve Bayes | -3.6210 | 0.0243 |
|  | Hypercholesterolemia |  |  |  |  |
|  |  | Decision tree | Discriminant analysis | 4.5139 | 0.0490 |
|  |  | Decision tree | KNN | -4.9104 | 0.0278 |
|  |  | Decision tree | Naïve Bayes | -3.9681 | 0.1006 |
|  |  | Discriminant analysis | KNN | -9.4243 | <.0001 |
|  |  | Discriminant analysis | Naïve Bayes | -8.4820 | <.0001 |
|  |  | KNN | Naïve Bayes | 0.9423 | 0.9427 |
|  | Current smoker |  |  |  |  |
|  |  | Decision tree | Discriminant analysis | 2.7779 | 0.6307 |
|  |  | Decision tree | KNN | -7.3412 | 0.0151 |
|  |  | Decision tree | Naïve Bayes | -9.0277 | 0.0020 |
|  |  | Discriminant analysis | KNN | -10.1191 | 0.0005 |
|  |  | Discriminant analysis | Naïve Bayes | -11.8056 | <.0001 |
|  |  | KNN | Naïve Bayes | -1.6865 | 0.8851 |
|  | Body Mass Index |  |  |  |  |
|  |  | Decision tree | Discriminant analysis | 4.0676 | 0.4418 |
|  |  | Decision tree | KNN | -7.6884 | 0.0334 |
|  |  | Decision tree | Naïve Bayes | -8.4820 | 0.0160 |
|  |  | Discriminant analysis | KNN | -11.7559 | 0.0005 |
|  |  | Discriminant analysis | Naïve Bayes | -12.5496 | 0.0002 |
|  |  | KNN | Naïve Bayes | -0.7936 | 0.9910 |
